# Co-creating community wellbeing initiatives: what is the evidence and how do they work?

**DOI:** 10.1186/s13033-024-00645-7

**Published:** 2024-08-05

**Authors:** Nicholas Powell, Hazel Dalton, Joanne Lawrence-Bourne, David Perkins

**Affiliations:** 1 Independent researcher. Formerly Centre for Rural and Remote Mental Health, University of Newcastle, Orange, NSW Australia; 2 Rural Health Research Institute, Charles Sturt University, Orange, NSW Australia; 3 School of Medicine and Public Health, University of Newcastle, Callaghan, NSW Australia; 4 Healthy Minds Research Program, Hunter Medical Research Institute, Newcastle, NSW Australia; 5 Mental Health Policy Unit, Health Services Research Institute, University of Canberra, Canberra, ACT Australia

**Keywords:** Community, Wellbeing, Mental health, Capacity building, Ownership, Co-design, Bottom-up, Collaborative

## Abstract

**Background:**

Addressing wellbeing at the community level, using a public health approach may build wellbeing and protective factors for all. A collaborative, community-owned approach can bring together experience, networks, local knowledge, and other resources to form a locally-driven, place-based initiative that can address complex issues effectively. Research on community empowerment, coalition functioning, health interventions and the use of local data provide evidence about what can be achieved in communities. There is less understanding about how communities can collaborate to bring about change, especially for mental health and wellbeing.

**Method:**

A comprehensive literature search was undertaken to identify community wellbeing initiatives that address mental health. After screening 8,972 titles, 745 abstracts and 188 full-texts, 12 exemplar initiatives were identified (39 related papers).

**Results:**

Eight key principles allowed these initiatives to become established and operate successfully. These principles related to implementation and outcome lessons that allowed these initiatives to contribute to the goal of increasing community mental health and wellbeing. A framework for community wellbeing initiatives addressing principles, development, implementation and sustainability was derived from this analysis, with processes mapped therein.

**Conclusion:**

This framework provides evidence for communities seeking to address community wellbeing and avoid the pitfalls experienced by many well-meaning but short-lived initiatives.

**Graphical Abstract:**

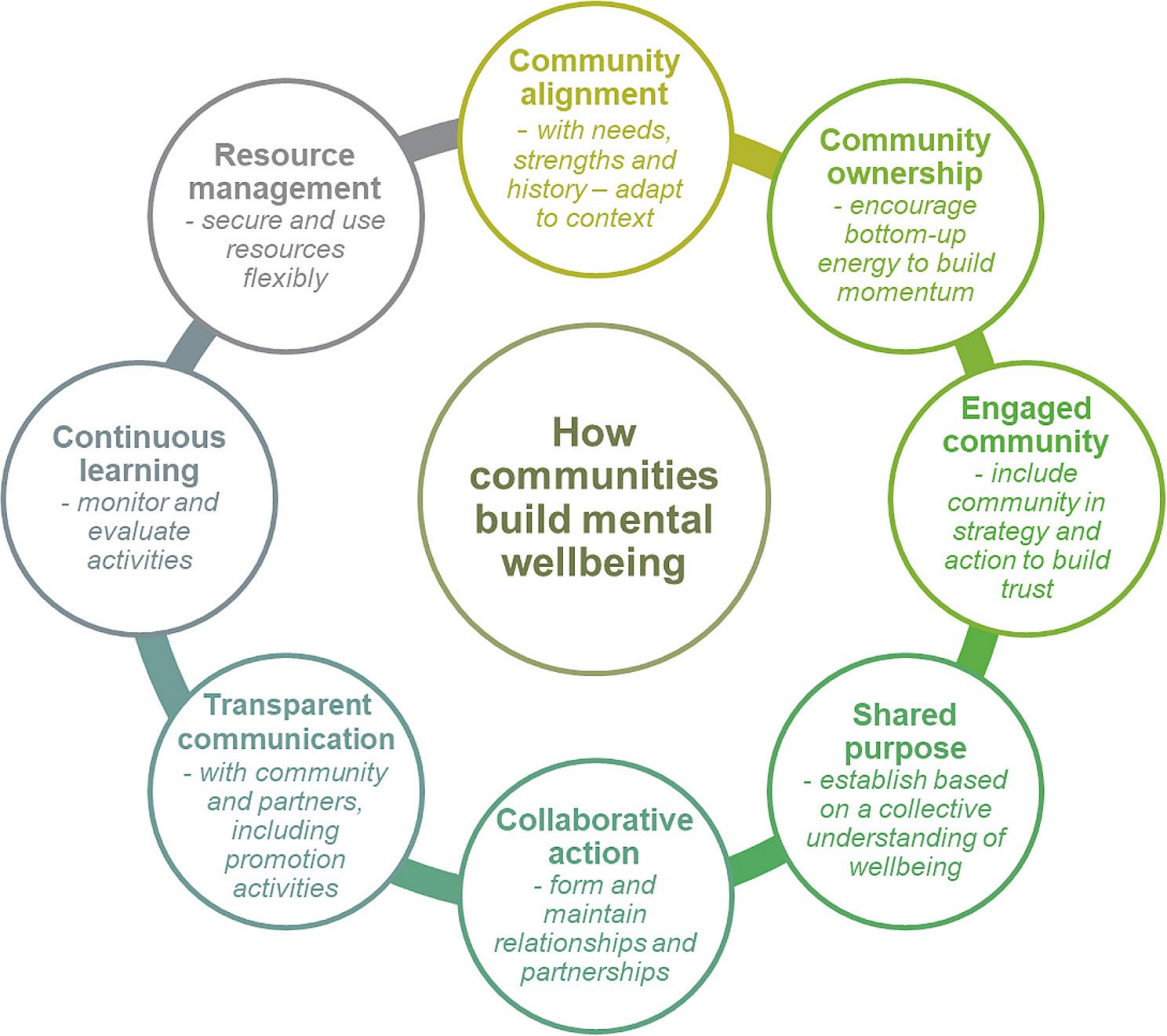

**Supplementary Information:**

The online version contains supplementary material available at 10.1186/s13033-024-00645-7.

## Background

Despite large investments, rates of mental illness have risen for decades, particularly in western societies [1]. The reasons are varied and complex and have been the subject of intensive study [2]. Risk factors include loneliness, inequality, disempowerment, and multiple and compounding adversities, factors that have been amplified by the COVID-19 pandemic [3]. There is widespread recognition of the problem and broad agreement that policy changes are needed [4, 5]. Recovery from the pandemic and an increased focus on mental health represent an opportunity to reinvent the way society invests in mental health and wellbeing. The question becomes where and how to intervene to improve population mental health and wellbeing.

Currently, most mental health expenditure occurs in the mental illness treatment system [4]. These individualised and medicalised approaches are effective for treating mental illness in some people [6, 7], but do little to prevent declining population mental health, promote wellbeing, or improve the conditions that contribute to mental illness [8]. It is more expensive and difficult to treat advanced illness than to intervene early or prevent the illness in the first place, thus prevention and early intervention activities are key components within a comprehensive mental health system [9]. The concept of ‘mental health’ has become stigmatized, associated with mental illness and mental health problems in the public eye [10], thereby limiting its utility in the promotion of positive mental health. Thus for the purpose of this review, we have used the concept of eudemonic wellbeing to indicate positive mental health. This is a process of living well that supports positive psychological and physical wellness, underpinned by a theory of self-determination [11].

Since the 1960s programs have been developed to empower or share power with communities to create social and health change [12]. These include the Alma Ata Declaration [13, 14], the Ottawa Charter [15], Healthy Cities [16], community empowerment projects [17–19], social capital promotion [20, 21] and action on the social determinants of health [22, 23], and have each made impacts on human wellbeing and informed the way that public and population health interventions are conducted [24]. Short political timeframes mean interventions in particular places are often abandoned, then replaced, this loss of continuity impairs trust and limits effectiveness [25]. Working through and with communities may be the most effective way to achieve long-term, independent and sustainable change, particularly when behaviour change is needed [26]. Initiatives to create vibrant and social communities may act at an appropriate level to improve mental health and wellbeing for all [27]. Working at the community level may also help to reframe the popular understanding of wellness so that poor wellbeing is seen less as a personal failing and more as a product of a pathogenic environment [28].

While there are many models of what community initiative can do to build wellbeing, there is little information on how they can accomplish these steps, or how factors change over time. For example, a review of community coalition-driven initiatives found beneficial changes in health outcomes and behaviours, however, there was insufficient process evidence on how the effects were mediated [12]. Previous research suggests that community level work should take a grassroots, bottom-up and codesigned, and collaborative approach (variously referred to as partnerships, coalitions, teams or working groups) that acknowledges complexity, power inequalities and shared priorities [29]. Community collaborative group-based social ecological approaches have been used for decades to facilitate ownership in a context-based and culturally sensitive manner through capacity building [30]. To help engage the community, there are framing and language recommendations so that needs and objectives are understood as opportunities, not problems or vulnerabilities [31, 32]. Top-down and overly medicalised models have been seen to fail [33]. The collective and relational nature of problems/assets such as loneliness/social capital and various social determinants of mental health reinforce the utility of a broader community focus on mental health and wellbeing.

### Community wellbeing initiatives

In this review, we examine the broader concept of community wellbeing initiatives by exploring the underlying sub-concepts. Wellbeing has become a catch-all term that is often used interchangeably or in partnership with mental and physical health, happiness, life satisfaction and others.

Whilst wellbeing is a more positive concept than mental health, it is a contested concept [34], used in communities, industry, policy and practice. Wellbeing has multiple aspects including: physical, mental, intellectual, social, emotional and spiritual components. As discussed above, in this review, we use the wellbeing definition as postulated in Ryan’s theory of self-determination, which stands in the positive mental health domain [11]. This distinguishes it from other community health and wellbeing reviews that focus on physical activity and dietary interventions.

In the context of public health and health policy, ‘community’ can be difficult to define. Two key approaches highlight geographical and functional communities [35]. This study uses the definition of “community to refer to a geographically bound group of people on a local scale who are subject to either direct or indirect interaction with each other” [36]. This is a setting where local place-based resources can be found and applied. Much policy focus has been applied to geographically bound areas, e.g. the UK government national approach is still deployed at the local government area level, where local context can be addressed [37]. Moreover, place is where things happen, such as natural disasters, acute economic insults such as the closure of local enterprises, and suicide clusters [3]. Place is where the context can be understood, challenges collectively felt and local strengths recognised and mobilised. Initiatives in these settings present an opportunity to deliver wellbeing and mental health promotion activities that are not provided by local health services, who focus overwhelmingly on the treatment of acute mental illness.

Community wellbeing concerns those factors that enable or hinder a citizen’s ability to build and maintain their wellbeing in a particular place [38–40]. For example, social capital, goods, infrastructure and service accessibility and cultural values influence individual wellbeing and can be built at the community level [41]. Community is where people live, it is the environment that shapes their wellbeing. In short, “community” concerns the level of analysis and “well-being” describes the scope of analysis [36].

In this paper, we analyse the literature on community-built wellbeing initiatives that have mental health and wellbeing as a stated objective or key outcome. For reasons discussed above, this paper focuses on initiatives that empower the community to create the change they wish to see in their area. The identified exemplars have been subjected to detailed analysis to create a common framework for the process factors associated with community wellbeing initiatives. The purpose of the study is to assist communities to build their own interventions to address mental health and wellbeing. The research questions are as follows:

1. Can community wellbeing initiatives, with wellbeing as a stated objective, be identified that have some implementation success as measured by (i) duration of existence (at least two years) and (ii) have published evidence regarding the initiative (e.g. peer-reviewed article)?
2. For the chosen community wellbeing initiatives:
  - What was the context for initiation?
  - Which stakeholders were involved and what were their roles in the successful implementation?
  - What was done to promote community wellbeing by these initiatives?
  - How was momentum sustained and progress measured?
  - What were the implementation and outcome lessons that may be used by other communities?

## Methods

### Search strategy

An initial scoping review of the field of community wellbeing [42], informed a structured search strategy, which was refined to remove false positives (excess unrelated papers). The search strategy focused on three factors: what were initiatives trying to achieve (what), the approach or philosophy they followed (how), and the area in which they worked (where). Results were limited to 2000–2019. The search strategy was developed in Medline and adapted for CINAHL, Web of Science, Psychinfo, Sociology Source and Cochrane library. The initial search strategy was deliberately broad to encapsulate the diversity of terms commonly used in this field. The search terms used, in Boolean structure of *What* AND *How* AND *Where*, were: ([wellbeing OR well-being OR mental health OR social determinant OR flourishing OR resilience OR social capital OR social cohesion OR salutogen* OR positive psychology] AND [ecological approach OR grassroot OR community driven OR capacity building OR empowerment OR engagement OR collective impact OR community development OR public health] AND [communit* OR local OR neighbo* OR city OR town]).

### Inclusion and exclusion criteria

The inclusion and exclusion criteria were refined via reflective collaborative discussion. Evidence type was restricted to primary papers to enable a primary analysis of process themes for this review. This included process and outcome evaluations. Commentaries and secondary analysis or theoretical papers were excluded but reserved for consideration for inclusion in introduction and/or discussion. Community settings were included, whilst papers focused on more restrictive settings such as school, prisons and aged care were excluded. Initiatives required community involvement and a wellbeing focus. Papers were excluded if the focus was not wellbeing, if it was clearly top-down, externally applied or if there was insufficient detail to describe the initiative activities, processes and governance.

### Data extraction, analysis and synthesis

Data extraction: All authors designed an analytical framework from which the data extraction tool was developed (Supplementary Table S1). Two authors (NP and JLB) reviewed each included paper using the data extraction tool to create a comprehensive dataset. Details of the initiatives were summarised and encompassed key details, formative and process factors.

A combination of content and thematic analysis [43] was used to identify themes and concepts within the dataset. The content analysis mapped to existing theories [17, 44–46] of community health initiatives to develop themes on the factors that contributed to the functioning of the initiatives. The processes of the initiatives were thematically grouped, coded and discussed by the authors until a coding framework of eight themes was developed, with sub-coding within a matrix to illustrate developmental stages over time. This was developed iteratively with author discussion and regular comparison to the twelve exemplar initiatives and primary themes (NP, HD, DP). Each stage of the thematic analysis was conducted by at least two authors. NVivo was used to organise the themes and the included papers were reanalysed against the coding framework.

## Results

### Search results

**Fig. 1:**
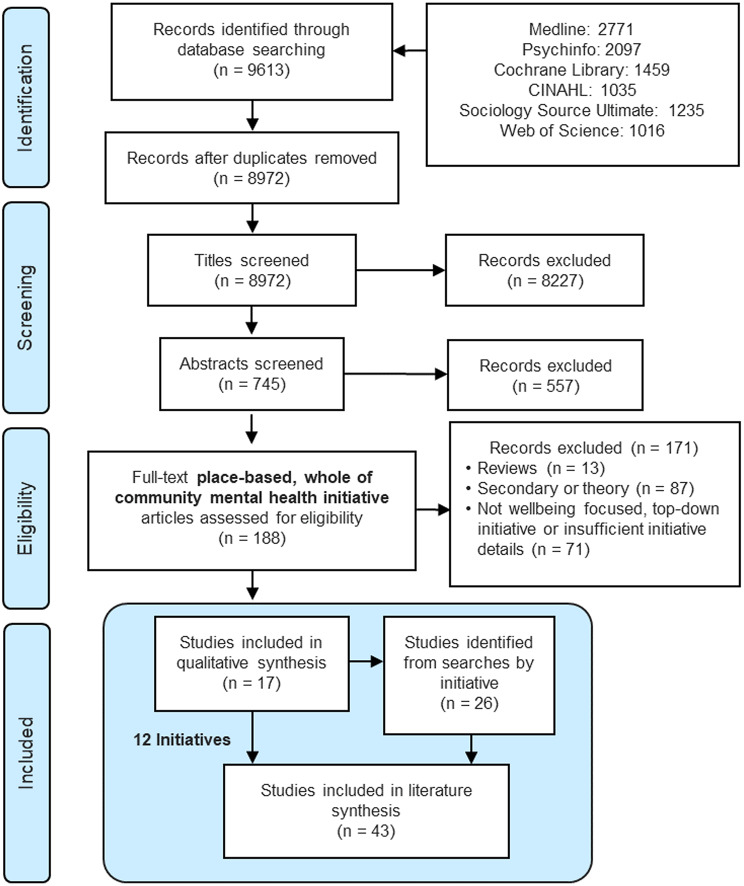
Preferred Reporting Items for Systematic Reviews and Meta-Analysis (PRISMA) four-phase flow diagram

The search returned 8972 results without duplicates. Title and abstract screening excluded 8784 records. Following the search strategy (Fig. 1), two authors (NP and JLB) read half of the papers each and compared notes. Disagreements were resolved in discussions with a third author (HD). A total of 17 papers describing twelve separate initiatives were identified. Google Scholar was searched for all literature related to these twelve initiatives, with 26 additional papers found, primarily related to two initiatives.

### Context of the initiatives

The twelve exemplar initiatives employed different approaches and came from numerous countries – one each from New Zealand, Canada, Mexico, the United States of America (USA), two from Australia and six from the United Kingdom (UK). Key details of these initiatives are summarized in Table 1.

**Table 1:** Summary of included exemplar initiatives

Initiative & Purpose	Location, Timeframe	Governance	Activities	Community role/s	Related papers
**1. Ranui Action Project** Improve health and wellbeing inequities of the Ranui people.	Auckland, New Zealand. 2001-ongoing^i^	Residents steering committee and local government.	Visioning and consultation sessions with formative evaluation specialist and established clear links between objectives, strategies, and activities. Wide-ranging program of activity targeting social capital and capacity building including: gardening projects, youth development camps, computer skills training, and driver license training.	Bottom-up self-governing entity. Local community members were early, active participants – involved with negotiations with the funders and fund holders.	[47]
**2. Headwaters Communities in Action** Promote a vigorous, sustainable and resilient community. Bringing different community sectors together to create solutions to shared problems and pursue creative opportunities together. Act as a catalyst to support collaborative projects to take root.	Ontario, Canada 2004-ongoing^i^	Leadership team of residents.	Developed a shared vision which was endorsed through community consultation with 350 residents. Produced an area wellbeing report with professional assistance. Promoted volunteerism, active transport, service innovations and sustainable farming practices. Administered community grants.	Representatives on the leadership team. Consult and endorse vision and implementation plan. Participate in working and project groups.	[48]
**3. Amigas Latinas Motivando el Alma** To build coping skills and resiliency, and address language barriers through academic partnership.	North Carolina, USA Unclear timeframe	University grant partnership.	Training of champions (promotoras).	Participate in community advisory community. Help develop plans for services and research.	[49–50]
**4. Well London** To promote healthier lifestyles (healthy eating, physical activity and mental wellbeing) among some of London’s poorest neighbourhoods.	London, UK 2007-ongoing^i^	Local steering groups oversaw the programs, neighbourhood advisory groups facilitated ongoing engagement.	Facilitation and coordinator training, World Café method to engagement, appreciative inquiry workshop, developed theory-of-change model, one-off and short-term events for behaviour change, educational courses, social capital buildings groups. Resident interviews, and evaluation. Activities that were in touch with local needs did well.	Neighbourhoods were selected by a governing body (Big Lotto) and community members became leaders and volunteers.	[55–68]
**5. Buen vivir** Create processes of change by stressing the importance of social context, culture, and local knowledge and enabling local enterprises to be socially entrepreneurial.	Chiapas, Mexico Unclear timeframe	Participatory governing bodies - Each Indigenous community ran their own.	Pursuit of social goals rather than profit-maximisation. Fair prices for goods.	Participate in leadership groups (the social enterprise model has participatory governance with community). Self-determine the social goals to work towards under *buen vivir.*	[69, 70]
**6. Priority Driven Research Partnership** To help achieve creative and mutually empowering ways for collaboration in two Indigenous communities.	Far North Queensland, Australia Approx. 2007–2011	Organised by research partnership.	Development of women’s and men’s groups to build empowerment. Especially through carer and consumer groups. Principles: empowerment and strengths-based approaches among community organisation to support better outcomes of consumer, families and communities.	Citizen leadership through recruitment of community health workers and community leaders /representatives.	[71]
**7. Community and Wellbeing Champions** Build knowledge about mental health and wellbeing through community capacity building in community champions.	London, UK 2012-2013	This is a part of a larger initiative that is delivered by an NGO. Champions were appointed by them.	Recruiting champions. Training and support for champions. Champions operated in their existing social circles: faith-based organisations were a focus. They tried to start conversations about mental health. Champions had meetings and events before committing to the role. Evaluations by researchers.	Champions were part of the communities. Began with community engagement with varied strategies for broad reach. This developed into partnerships and a shared vision.	[72, 73]
**8. Big Local** To empower communities to address health inequalities. To build collective control to address health and wellbeing in communities. Collective control has been linked to mental health.	UK 2010-ongoing^i^	Funded by the national lottery. Local partnerships were developed and supported in selected communities to support leadership.	Process was partnership formation, consultation with the broader community, creation of the delivery plan, endorsement by the Local Trust (organising body/charity) and then implementation. A wide variety of smaller partnership activities were organised (fashion shows, dog shows, cooking events, growing/planting projects, family fun days and galas, music and dance performances and community arts projects). Often these events were designed to engage with marginalised groups. Appointment of volunteers. Advice from experts.	The local partnerships that were formed were responsible for developing and delivering the program. They developed shared vision and priorities. Partnerships had to be at least 51% residents.	[74–79]
**9. Our Healthy Clarence** A community-driven strength-based approach to wellbeing promotion and, by extension, suicide prevention, including positive health promotion, primary and secondary prevention activities, advocacy, and cross-sectoral collaboration.	Clarence Valley, New South Wales, Australia 2016-ongoing^i^	Steering group of local residents, service providers and community leaders, and a local coordinator.	Community workshops, shared vision creation and plan development. Working groups to implement strategies. Advocate to government. Community activities and events under the banner of the initiative. Foundation of hubs for meeting and service provision/linking.	Leadership and participation in the steering and working groups. Consultation in formation of community plan.	[80]
**10. Transition Town Totnes** Improve sustainability of the town in terms of climate change, economy and socially.	Totnes, UK 2006-ongoing^i^	Small street communities that meet to create opportunities and activities.	Activities are designed around a group of themes: Arts, Food (e.g., Food hubs to bring people together), Building, Housing and Energy, ‘Reconomy’, Inner Transition, Skillshares (knowledge sharing), Transportation, transition Streets, Play and Education. Media messaging and marketing. Had their own currency.	Created and led by local residents to work on their town. Appears to be a truly bottom-up initiative that arose without top-down funding or support.	[81, 82]
**11. Altogether Better: Community Health Champions** To empower people across the Yorkshire and Humber region to improve their own health and that of their families and their communities.	Yorkshire and Humber, UK Approx. 2006-ongoing^i^	Part of a larger initiative (National lottery funded). Champions were recruited and supported by it, with a ‘light touch’.	Recruitment and training of champions. Health champions: • Lead organised health walk • Work in allotment and food growing initiatives • Set up social clubs • Deliver health awareness presentations on chronic conditions • Signpost locals to relevant services and resources.	Most champions came from the communities in which they were appointed and became leaders for change there.	[83, 84]
**12. Happy City** Create a real-world, engaged, bottom-up approach to happiness in a community.	Bristol, UK 2009-ongoing^i^	Led by a newly created not-for-profit organization.	Communication campaigns with strengths-based language. Partnership building through events. Workshops on wellbeing. Became an enabler of change for wellbeing. Developed a set of tools to assist individuals, communities and policymakers to evaluate and improve wellbeing.	The people running the initiative came from the community. Community members were set up to buy into and receive help from the initiative.	[85]

^i^-Ongoing at time of review

### Wellbeing approaches of the initiatives

To promote mental health and wellbeing, all initiatives encouraged the social dimensions of community, working to build social capital and many using community champions (initiatives # 3, 7, 11) and encouraging volunteerism (initiatives #2, 8, 9, 10). Typical health promotion activities were commonly used, including training (initiatives #3, 7, 9, 11, 12), raising awareness, de-stigmatising conversations, encouragement of self-reflection on what wellbeing and resilience meant to individuals, use of campaigns and tools such as ‘Five ways to wellbeing’ [86] (initiative #12) and providing opportunities for safe and social interactions (pop-up hubs, youth spaces, and community events). Others explicitly recognised the social and economic determinants of health and were also addressing those (initiatives #1, 2, 5, 10).

### What assisted the initiatives to function?

**Fig. 2:**
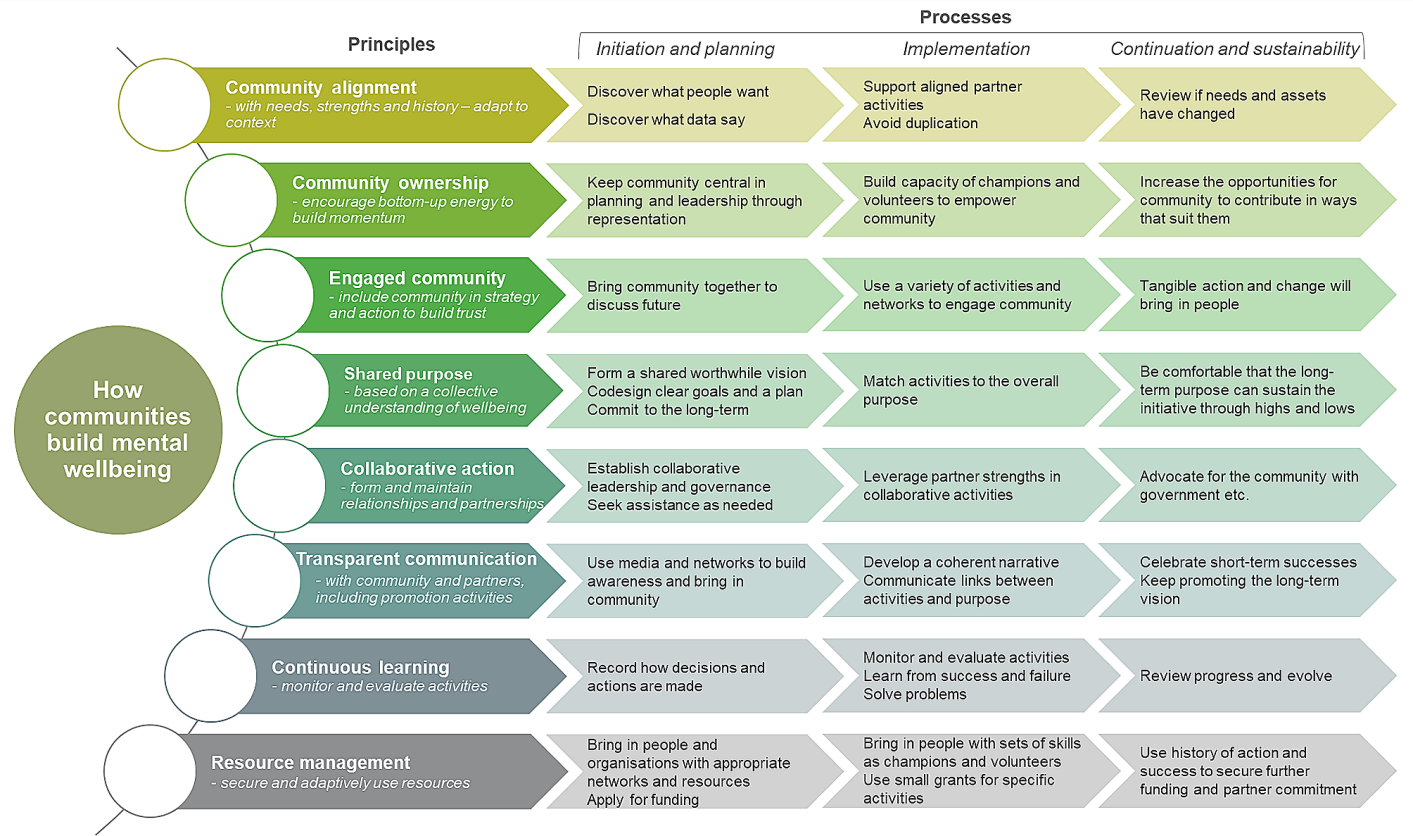
Framework for community wellbeing initiatives – key principles and processes

We found eight key themes associated with successful community mental wellbeing initiatives (summarised under ‘principles’ in Fig. 2). The way that communities understood and exhibited each of these themes changed over time. For example, initial community engagement often focused on gathering community opinion and later developed into planning events and participation in working groups. The stages of the initiatives are iterative and there was no consistent developmental process – therefore these stages are more a set of component processes to be developed and monitored, rather than a definitive sequence. Each of these broad themes was found in at least eleven of the twelve initiatives and can be thought of as principles that underpinned the initiatives. Moreover, these principles were operationalised as different processes at different phases (‘initiation and planning’, ‘implementation’ and ‘continuation and sustainability’) and are summarised in Fig. 2. The coding references for these principles and processes mapped against each initiative can be accessed in Supplementary Table S2.

### 1. Community alignment – align with community needs, strengths, and history – adapt to context

The community initiatives included were sensitive to the context in which they operated. During initiation and planning, the collection of subjective and objective data enabled a contextual understanding of community need. The general community was asked what they wanted to change (10 of 12 initiatives), and publicly available community data was reviewed (9 of 12 initiatives), highlighting community assets and helping to prioritise needs. As some of the initiatives began to put this information into action, they took care to not duplicate existing activities (2 of 12 initiatives), which can cause wasted energy, community confusion and detract from the credibility of and support for the initiative. As the initiatives matured it was important to consult the community regularly (4 of 12 initiatives) to ensure that the initiative was adaptive and responsive to changes.

### 2. Community ownership – encourage bottom-up energy to build community ownership

The first step to generate community ownership, was to keep the community voice and vision as the anchor point for all planning and leadership (8 of 12 initiatives). This was achieved through community representation, but some initiatives navigated the concept of representation, with particular representatives having multiple roles. For example, if a professional member of the leadership group was appointed to represent their organisation, could they also be a resident representative? This raised considerations of conflicts of interest and how to handle them. Secondly, the ideas of empowerment and ownership are entwined. Capacity building of champions and volunteers were considered important steps to making their initiatives more acceptable and sustainable in the community (9 of 12 initiatives). Giving the community flexible opportunities to contribute to the initiative in ways that suit members was also important (8 of 12 initiatives). This allowed community members to “dial in and out” of the initiative depending on their interests and commitments.

### 3. Engaged community – include community in strategy and action to build trust

Five initiatives brought the community together to discuss the future, which was key to their planning and visioning, and may have played a role in engaging community members in leadership or working group positions. Diverse combinations of activities and networks were used to engage with a broad range of community members (11 of 12 initiatives). This is a recognition that not all community members can be reached through traditional networks and that not all activities will engage all community members. It was recognised that in the long term, tangible action and change in the community were key to engaging more people (3 of 12 initiatives).

### 4. Shared purpose – establish based on a collective understanding of wellbeing

A shared vision that reflected the community voice and aligned with the local context was an important factor in the organisation of initiatives (11 of 12 initiatives). Since wellbeing is a subjective term for individuals and communities, the visions were often based on a local understanding or definition. The desire to create an agreed community vision was undercut by concerns that many initiatives developed a vision based on influential, generally upper middle-class concerns of a subset of the community, rather than being truly representative. The shared visions were translated into specific goals or plans by at least nine of the initiatives. The importance of committing to the long term was raised, since the desired social change could not be achieved in the one to two years that were commonly funded (5 of 12 initiatives). To keep initiatives on track, the consistent linking of activities back to the overarching purpose helped get community involved and keep the leadership and working groups motivated (6 of 12 initiatives). Three initiatives recognised that the human value in the purpose of their initiative helped sustain the initiative through challenges.

### 5. Collaborative action – form and maintain relationships and partnerships

The selected initiatives had a locally based, collaborative leadership team (12 of 12 initiatives). The ways in which these teams arose differed, with some aided by an external organisation visiting the community and assisting in building a community leadership group (2 of 12 initiatives), others formed leadership groups as a result of local energy (3 of 12 initiatives), although they were assisted by external support to establish and legitimise their initiative.

Collaborative action was evident (11 of 12 initiatives). This included collaboration between community members, local council, health and mental health services, the education system, law enforcement, researchers, local businesses, and voluntary organisations. Many described the formation of a collaborative leadership and governance structure in the form of a steering committee (9 of 12 initiatives). The importance of partnering and supporting relevant community activities was outlined (6 of 12 initiatives). Assistance was sought for certain activities, including workshop facilitation, needs assessments, obtaining funding and evaluation (10 of 12 initiatives). Some of this external support also relied upon government intervention, especially on the issues that cannot be addressed by a community initiative. On these issues, some of the initiatives advocated to government, rather than assume responsibility for endemic issues (e.g., poor employment opportunities, housing or recreation space).

### 6. Transparent communication – openly communicate with community and partners, including promotion activities

Active communication between the initiative and the broader community was used to engage the community for initial discussion; to involve members as leaders, volunteers, or champions to advertise the purpose and vision of the initiative; to publicise the plan; to advertise sponsored or organised activities; and to list key community contacts for support or involvement (8 of 12 initiatives). This was achieved through promotion in traditional and new media (9 of 12 initiatives), through established networks and word of mouth. Communication between members of the initiative was important for cohesion and enabling democratic elements of decision making (8 of 12 initiatives). Development of a coherent narrative was key to the overall communication and engagement strategy (6 of 12 initiatives). Consistent explanation of the link between the activities of the initiative and the overall purpose was valued (8 of 12 initiatives). Celebrating short-term successes and promoting the long-term vision can illustrate that worthwhile change is possible and occurring (7 of 12 initiatives).

### 7. Continuous learning – monitor and evaluate activities

Each initiative adapted over time as they learned how to operate and be effective (12 of 12 initiatives). Continuous learning and improvement through monitoring and evaluating activities was described (11 of 12 initiatives). In the organisation stage, some made a point to record how decisions and actions were planned (4 of 12 initiatives). As the initiatives were implementing activities, solving problems and learning from success and failure were key parts of the initiative’s maturation (8 of 12 initiatives). To work towards sustainability in their community, progress reviews were central (11 of 12 initiatives) and helped initiatives to evolve as community needs and assets changed.

### 8. Resource management – secure and use resources flexibly

Several of the initiatives were described from the perspective of the funders, making it challenging to assess the financial resource dimension. Some initiatives were established only as funding was secured; others secured funding as they went along. Funding was often discussed, including receipt or application for funding and how relationships with funders were managed (7 of 12 initiatives). Small grants for very specific activities, often short term, were easier to obtain in some communities (5 of 12 initiatives). The gathering of non-fiscal resources was discussed by more initiatives than fiscal ones (10 of 12 initiatives). The importance of bringing in organisations and people with the networks and resources to support the initiative was identified, particularly in the early stages (9 of 12 initiatives). While networks and resources were particularly important in the planning stage, people with particular skills who could act as leaders, champions and volunteers were valuable in implementation and maturation. Finally, the importance of a history of action and success in securing new resources was discussed (2 of 12 initiatives). Grant applications were more successful if the initiative demonstrated a strong track record. Also, local organisations and individuals were more likely to contribute towards the initiative when they see that it is a realistic pathway to change.

## Discussion

In this study, we have sought to identify the principles and processes employed by successful community led initiatives to improve mental health and wellbeing. Success in this case was measured by duration of initiative (greater than two years) and supportive evidence of the initiative’s process development or outcomes in the literature. Twelve exemplar community-built wellbeing initiatives were identified. From these, detailed analysis yielded eight key themes (principles) associated with the factors that contributed to the functioning of the initiatives. These principles were community alignment, community ownership, community engagement, shared purpose, collaborative action, transparent communication, continuous learning, and resource management. These were expanded into a matrix to illustrate how the principles were enacted in practice (processes) over the developmental stages of initiation and planning, implementation, and continuation and sustainability. Thus, there is a matrix of component processes associated with how these initiatives were able to collaboratively address mental health and wellbeing in their communities in response to local need and with local ownership. These may be of interest and use for other collaborative initiatives aimed at addressing wellbeing.

Wellbeing is a complex phenomenon, which the twelve exemplar initiatives addressed multidimensionally, deliberately and contextually. The community alignment principle was exemplified by capturing community needs and strengths, both subjectively by listening to community members and objectively via public data sources, e.g. [80]. These included activities to enhance social connection, increase volunteering, access to support, access to leisure and hobbies, access to green and blue spaces and other social determinants of health that are supported in broader research [12, 41, 87–90]. Activities were chosen in response to community aspirations, the shared purpose, building both community ownership and engagement. Extensive community involvement in the initiatives was evident, providing information regarding need, collaborative planning and action, leveraging with partners and engaging with the wider community. This aligns with the weight of evidence which suggests that community codesign, empowerment or ownership are strongly linked to success, effectiveness and sustainability of health and behaviour change initiatives [91].

Community ownership was found to be a key principle; however, the evidence also suggested the importance of external support which could enable complex change in a community (bridging the principles of resource management and collaborative action). Therefore, some level of authority or decision-making power contributing top-down (outside-in) support in combination with bottom-up (inside-out) support and energy are essential for sustainability [92, 93]. This raises an important point about power management within community initiatives [94]. The findings presented here support the broader literature, suggesting that the role of authority figures is to enable and aid the community to realise the codesigned vision [92, 95]. The general recommendations from community public health initiatives are to enable a community to contribute and develop agency, that is, health interventions done with communities, not to communities [91, 96–102]. As such, when the goals of an initiative are closely aligned with those of local government, cross sector collaboration flourishes (community alignment and collaborative action principles) [103–105].

Most of the community initiatives operated at a level between local government and the public. While power management was a consideration for most, the question of capacity and capabilities (resource management) was also relevant in deciding upon vision, goals, objectives, responsibilities and contributions [80, 106]. Every community has local organisations with capacity to enact change at some level within the community. Whilst no single partner organisation might be essential for community initiatives, each partner opens new opportunities, and these partners may influence both ambitions and the ability to realise them (collaborative action). In order to understand community change, we must acknowledge that there are other networks, structures and systems that influence and can be leveraged to influence the overall outcome [107, 108].

Transparent communication was a key principle, and was used by the included initiatives to traverse the developmentally vulnerable stage of initiation and planning [109]. Communication of short-term achievements and celebrations was used to build momentum, grassroots support and help reinforced a sense of realistic expectations. Active communication strategies can help build a coherent narrative and link activities to the shared purpose. However, for true change, the many factors that influence wellbeing in the community must be improved upon and be seen to be improving. There is evidence that citizens perceptions of their community and their pride in community are closely linked to the way they talk about their life satisfaction and mental wellbeing, and may be a key pragmatic measure for initiative success [78, 110, 111].

The challenges of evaluating the success of community-based initiatives persist [112], with attribution of causation especially difficult [113, 114]. As noted earlier, a Cochrane review of community coalition-driven interventions [12] found evidence for positive benefit to individual health outcomes and behaviours, and care delivery systems. There was insufficient evidence on the workings of the coalitions themselves to explain how benefits were achieved, indicating a gap in process evidence. Whilst capturing both the process and the outcome is valued by the researcher, consideration should be given to the burden of documentation in a community-driven initiative should be given. There is a delicate balance between maintaining formal mechanisms and processes to track an initiative without intimidating or overpowering community voice and resourcefulness [114, 115]. This study highlights that the documentation of initiative processes and activities supports three of the eight principles directly: the ability to share information and build the common narrative (transparent communication), to reflect and learn (continuous learning), and to leverage future funds with evidence of activity and impact (resource management). This may be a valuable strategy since short, fixed-term funding was identified as a key challenge for resource management and sustainability, which can lead to diminished trust for future initiatives [116, 117]. By addressing documentation and evaluation of processes, activities and outcomes, further funds and resources may be secured, thus, providing the time needed to build and retain trust in the community wellbeing initiative. In recognition of the persistent challenge of short-term funding, contrasted with the acknowledged need for time to build trust and genuine collaborative action, policymakers and funders need to adjust to longer time spans. There are some relatively new philanthropic initiatives with explicitly longer time horizons of 10 or more years to address this and to genuinely work with communities for the duration of investment. These include the Hogg Foundation’s Collaborative Approaches to Well-Being in Rural Communities program in Texas [118], and the Fay Fuller Foundation’s Our Town program in South Australia [119]. These new funding approaches remain the exception, not the norm.

### Strengths and limitations

Strengths: This study has focused on the processes of how community wellbeing initiatives develop and function, where most papers focus on short-term outcomes. It has covered a wide range of papers written by academics and others from a variety of disciplines using a variety of similar, overlapping, and distinct terminology. We have attempted to address temporal issues recognizing that needs for leadership, resources and engagement vary over time and due to changing circumstances. We have suggested a common language/framework which is accessible to communities, not just community development or other professionals.

Limitations: We could only analyse what has been published which is limited in many ways. Initiative processes are often poorly described or assumed, important components are missed out, and papers are written from particular perspectives or with partial perspectives. Our included initiatives had variable volumes of evidence with some having one paper, others many, and up to 14 papers for Well London. Some initiatives are not described or published and therefore there is likely a lot we don’t know. While considerable effort was made to find and select appropriate materials, we may have missed something. There is no common measurement of outcomes, although the variety of contexts may invalidate such comparisons [120]. Moreover, due to the varying reporting on outcomes, our criteria for selecting successful cases was limited to implementation success, as measured by (i) duration of existence (at least two years) and (ii) have published evidence regarding the initiative (e.g. peer-reviewed article). We also note that the initiatives included all come from Western democracies, predominantly English-speaking countries, and thus the process characteristics outlined here may not apply in other national and cultural contexts.

## Conclusion

This review took a rigorous approach to finding twelve exemplar communities, which had successfully implemented community wellbeing initiatives. The focus on how the initiatives were implemented and sustained should aid interested communities to grow their own initiatives and may be used by other studies to design projects that can assess success and impact.

### Electronic supplementary material

Below is the link to the electronic supplementary material.

Supplementary Material 1

Supplementary Material 2

## Data Availability

Not applicable.
