# Supplementary material for: Co-creating community wellbeing initiatives: what is the evidence and how do they work?
Source: Int J Ment Health Syst. 2024 Aug 5;18:28. doi: 10.1186/s13033-024-00645-7 (PMC11299278; doi:10.1186/s13033-024-00645-7)
Supplement: Supplementary file 1 — Supplementary Material 1 [file 13033_2024_645_MOESM1_ESM.docx]

**Table S1.** Data extraction tool

| **Data extracted** | **Details** |
| --- | --- |
| Initiative | Name of the community wellbeing initiative |
| Author & study design | Year of publication, lead author and study details (e.g. summative evaluation). Repeated for multiple papers associated with the same initiative. References additionally captured in EndNote. |
| Location | Town or city, state if applicable, and country where the initiative took place. |
| Timeframe | Time frame for the initiative – start and finish year, including if ongoing at the time of review. |
| Motivation | Details the motivation or rationale for the initiative inception |
| Leadership and governance | Details of leadership and governance arrangements for the initiative |
| Degree of planning | Details of planning described in paper/s – e.g. workshops, community consultation summaries, plans |
| Implementation details | Details of the processes (how initiative was organised and run) and the activities of the initiative (what they did to promote wellbeing). |
| Community role/s | Specific details of how community members were involved (e.g. participation, representation on steering groups, volunteers and champions). |
| Purpose and outcomes | The stated purpose of the initiative and any details on outcomes |
| Strengths and limitations | Details of the strengths and limitations of the paper, both stated and interpreted (e.g. gaps in the ability of reviewers to fill out data extraction tool highlight limitations for the purpose of this review) |

^1^Missing data were coded as ‘not stated’.
