# Supplementary material for: Co-creating community wellbeing initiatives: what is the evidence and how do they work?
Source: Int J Ment Health Syst. 2024 Aug 5;18:28. doi: 10.1186/s13033-024-00645-7 (PMC11299278; doi:10.1186/s13033-024-00645-7)
Supplement: Supplementary file 2 — Supplementary Material 2 [file 13033_2024_645_MOESM2_ESM.docx]

**Table S2.** Principles and process^a^ themes with the number of associated coding references per initiative^b,c^

| **Initiative number** | **1** | **2** | **3** | **4** | **5** | **6** | **7** | **8** | **9** | **10** | **11** | **12** | **Total^d^** |
| --- | --- | --- | --- | --- | --- | --- | --- | --- | --- | --- | --- | --- | --- |
| **1. Community Alignment – Align with community needs, strengths and history, adapt to context** | 3 | 8 | 5 | 18 | 3 | 2 | 1 | 12 | 8 | 3 | 1 | 2 | 12/12 |
| IP - Discover what people want | 3 | 3 | 5 | 8 | 0 | 2 | 1 | 6 | 3 | 2 | 1 | 0 | 10/12 |
| IP - Discover what data and evidence say | 1 | 4 | 1 | 1 | 0 | 2 | 0 | 3 | 2 | 1 | 1 | 0 | 9/12 |
| I - Avoid duplication | 0 | 0 | 0 | 3 | 0 | 0 | 0 | 3 | 0 | 0 | 0 | 0 | 2/12 |
| C - Review if needs and assets have changed | 0 | 3 | 2 | 0 | 0 | 0 | 0 | 2 | 2 | 0 | 0 | 0 | 4/12 |
| **2. Community ownership – Encourage bottom-up energy to build community ownership** | 6 | 7 | 5 | 14 | 4 | 1 | 3 | 7 | 12 | 3 | 9 | 2 | 12/12 |
| IP - Keep community central in planning and leadership through representation | 4 | 6 | 2 | 8 | 2 | 0 | 0 | 2 | 7 | 2 | 0 | 0 | 8/12 |
| I - Build capacity of champions and volunteers to empower community | 2 | 0 | 2 | 7 | 0 | 1 | 3 | 0 | 4 | 1 | 8 | 2 | 9/12 |
| C - Increase the opportunities for community to contribute in ways that suit them | 0 | 1 | 1 | 2 | 2 | 0 | 0 | 6 | 1 | 2 | 1 | 0 | 8/12 |
| **3. Engaged community - Include community in strategy and action to build trust** | 3 | 4 | 3 | 6 | 0 | 1 | 6 | 6 | 6 | 10 | 5 | 1 | 11/12 |
| IP - Bring community together to discuss future | 0 | 1 | 0 | 2 | 0 | 0 | 1 | 0 | 2 | 2 | 0 | 0 | 5/12 |
| I - Use a variety of activities and networks to engage community | 3 | 3 | 3 | 5 | 0 | 1 | 5 | 5 | 3 | 7 | 5 | 1 | 11/12 |
| C - Tangible action and change will bring in people | 0 | 1 | 0 | 0 | 0 | 0 | 0 | 0 | 1 | 1 | 0 | 0 | 3/12 |
| **4. Shared purpose – Establish based on a collective understanding of wellbeing** | 8 | 16 | 2 | 8 | 7 | 3 | 2 | 6 | 10 | 6 | 3 | 2 | 12/12 |
| IP - Form a shared worthwhile vision | 2 | 10 | 1 | 3 | 4 | 1 | 1 | 3 | 6 | 4 | 0 | 1 | 11/12 |
| IP - Codesign clear goals and a plan | 3 | 3 | 1 | 2 | 2 | 2 | 0 | 3 | 2 | 2 | 0 | 0 | 9/12 |
| IP - Commit to the long-term | 3 | 3 | 0 | 0 | 0 | 0 | 0 | 1 | 1 | 0 | 3 | 0 | 5/12 |
| I - Match activities to the overall purpose | 1 | 1 | 0 | 2 | 2 | 0 | 1 | 0 | 4 | 0 | 0 | 0 | 6/12 |
| C - Be comfortable that the long-term purpose can sustain the initiative through highs and lows | 1 | 3 | 0 | 0 | 0 | 0 | 0 | 0 | 0 | 0 | 1 | 0 | 3/12 |
| **5. Collaborative action – Form and maintain relationships and partnerships** | 6 | 9 | 5 | 7 | 2 | 3 | 2 | 8 | 13 | 0 | 2 | 4 | 11/12 |
| IP - Establish collaborative leadership and governance | 5 | 2 | 5 | 1 | 2 | 3 | 1 | 3 | 5 | 0 | 0 | 0 | 9/12 |
| IP - Seek assistance as needed | 2 | 3 | 1 | 4 | 0 | 1 | 1 | 5 | 3 | 0 | 1 | 2 | 10/12 |
| I - Leverage partner strengths in collaborative activities | 0 | 0 | 0 | 1 | 1 | 0 | 0 | 1 | 3 | 0 | 1 | 2 | 6/12 |
| C - Advocate for the community to government etc | 2 | 3 | 1 | 4 | 0 | 1 | 1 | 5 | 3 | 0 | 1 | 2 | 10/12 |
| **6. Transparent communication – Openly communicate with community and partners, including promotion activities** | 3 | 7 | 2 | 4 | 0 | 1 | 5 | 12 | 7 | 4 | 4 | 3 | 11/12 |
| IP - Use media and networks to bring in community | 1 | 2 | 0 | 1 | 0 | 0 | 2 | 4 | 2 | 2 | 1 | 2 | 9/12 |
| IP - Communicate with and facilitate mutual support for leaders, partners, volunteers, champions etc. | 0 | 1 | 1 | 1 | 0 | 1 | 2 | 1 | 0 | 1 | 2 | 0 | 8/12 |
| I - Develop a coherent narrative | 1 | 4 | 0 | 2 | 0 | 0 | 0 | 3 | 3 | 0 | 1 | 0 | 6/12 |
| I - Communicate links between activities and purpose | 1 | 1 | 0 | 1 | 0 | 0 | 2 | 1 | 1 | 0 | 1 | 1 | 8/12 |
| C - Develop a community narrative and reputation by celebrating short-term success and promoting the long-term vision | 1 | 4 | 1 | 2 | 0 | 0 | 0 | 6 | 4 | 0 | 1 | 0 | 7/12 |
| **7. Continuous learning – Monitor and evaluate activities** | 3 | 10 | 4 | 13 | 1 | 1 | 5 | 7 | 6 | 0 | 2 | 2 | 11/12 |
| IP - Record how decisions and actions are made | 0 | 0 | 1 | 0 | 0 | 0 | 2 | 1 | 1 | 0 | 0 | 0 | 4/12 |
| I - Solve problems and learn from success and failure | 2 | 3 | 3 | 5 | 0 | 0 | 4 | 4 | 4 | 0 | 2 | 0 | 8/12 |
| C - Review progress and evolve | 2 | 7 | 1 | 7 | 1 | 1 | 1 | 4 | 1 | 0 | 2 | 2 | 11/12 |
| **8. Resource management – Secure and use resources flexibly** | 6 | 10 | 2 | 9 | 1 | 1 | 7 | 5 | 7 | 1 | 8 | 3 | 12/12 |
| IP - Bring in people and organisations with appropriate networks and resources | 1 | 4 | 0 | 3 | 0 | 1 | 2 | 3 | 3 | 0 | 1 | 1 | 9/12 |
| IP - Apply for funding | 2 | 3 | 1 | 2 | 0 | 0 | 0 | 1 | 1 | 0 | 1 | 0 | 7/12 |
| I - Bring in people with sets of skills as leaders, champions and volunteers | 2 | 1 | 1 | 4 | 0 | 0 | 5 | 1 | 2 | 1 | 5 | 2 | 10/12 |
| I - Use small grants for specific activities | 1 | 1 | 0 | 1 | 0 | 0 | 0 | 1 | 1 | 0 | 0 | 0 | 5/12 |
| C - Use history of action and success to secure further funding and partner commitment | 0 | 2 | 0 | 0 | 0 | 0 | 0 | 0 | 0 | 0 | 1 | 0 | 2/12 |
| 1. Key: IP = initiation and planning, I = implementation, C = continuation and sustainability. 2. Initiatives: 1. Ranui Action Project, 2. Headwaters Communities in Action, 3. Amigas Latinas Motivando el Alma, 4. Well London, 5. Buen vivir, 6. Priority Driven Research Partnership - Indigenous Australian Communities, 7. Community and Wellbeing Champions, 8. Big Local, 9. Our Healthy Clarence, 10. Transition Towns,11. Altogether Better Community Health Champions, 12. Happy City. 3. E.g., Principle 1, Community alignment, has 18 different reference codes associated with that theme from initiative #4 (Well London). 4. Total represents the total number of initiatives where the theme has been represented. E.g. Principle 1, Community alignment, has all 12 out of 12 initiatives with at least one reference code associated with that theme. | | | | | | | | | | | | | |
